# Identification of metabolites of dalfampridine (4-aminopyridine) in human subjects and reaction phenotyping of relevant cytochrome P450 pathways

**DOI:** 10.3109/21556660.2013.833099

**Published:** 2013-08-14

**Authors:** Anthony Caggiano, Andrew Blight

**Affiliations:** Acorda Therapeutics, Inc., ArdsleyNY

**Keywords:** Dalfampridine, 4-aminopyridine, Metabolism, Reaction phenotyping, Cytochrome P450

## Abstract

**Objectives:**

An extended release formulation of dalfampridine (4-aminopyridine; 4-AP), a potassium channel blocker is available in the USA to improve walking in patients with multiple sclerosis. This study investigated the human metabolites of 4-AP and the cytochrome P450 (CYP450) pathways responsible for 4-AP metabolism.

**Methods:**

Metabolites were identified, using thin layer chromatography, high performance liquid chromatography, and gas chromatography/mass spectroscopy, in plasma and urine samples obtained during an excretion balance study of four subjects who were administered a single oral 15-mg dose of ^14^C-4-AP. Samples were compared with authentic standards of 4-AP, 2-hydroxy-4AP, 3-hydroxy-4AP, and 4-AP-N-oxide. Reaction phenotyping was performed *in vitro* using human liver microsomes and recombinant CYP450 enzymes with incubation in the presence of direct and time-dependent inhibitors to determine the CYP450 pathways involved in metabolite formation.

**Results:**

While most (∼70%) of the radioactivity detected in plasma at each time point corresponded to unchanged 4-AP, two major metabolites were recovered. One metabolite co-localized with the authentic reference standard of 3-hydroxy-4-AP, and the other metabolite was identified as the sulfate conjugate of 3-hydroxy-4-AP. Two minor components were observed, one accounting for 2% of radioactivity and the other below the level of quantitation. Reaction phenotyping showed moderate correlations for conversion of 4-AP to 3-hydroxy-4AP with both CYP2E1 (*r* = 0.596; *p* < 0.001) and CYP2C8 (*r* = 0.608; *p* < 0.001). Use of a CYP2E1 metabolism-dependent inhibitor inhibited formation of 3-hydroxy-4-AP with and without pre-incubation (higher inhibition with pre-incubation), further supporting the likelihood of CYP2E1 as a metabolic pathway. The main limitation of this study was the inability to identify the CYP enzymes responsible for the 3-hydroxylation of 4-AP, although this conversion represents only a minor metabolic pathway.

**Conclusion:**

There is limited metabolism of 4-AP in humans. The two major metabolites were 3-hydroxy-4-AP and 3-hydroxy-4-AP sulfate, likely through CYP2E1 pathways; the possibility of other CYP enzymes playing a minor role in 4-AP metabolism could not be established unequivocally. Overall, these data suggest that there is a low risk for drug–drug interactions via an impact on 4-AP metabolism through cytochrome pathways.

## Background

Dalfampridine (4-aminopyridine; 4-AP) is a potassium channel blocker that has been approved by the United States Food and Drug Administration (FDA) as dalfampridine extended release tablets (dalfampridine-ER), 10 mg, for the treatment of walking impairment in patients with multiple sclerosis1. This formulation is known as prolonged-, modified, or sustained release fampridine in some countries outside of the USA. Approval was based on two phase 3 clinical trials that showed an approximate mean 25% increase in walking speed among the 35–43% of patients who responded to treatment with dalfampridine-ER2,3.

A review of the pharmacokinetics of dalfampridine-ER has recently been published4, and relative to immediate release dalfampridine, dalfampridine-ER is characterized by a longer time to maximum plasma concentration (3.2 vs 1.2 hours) and a longer apparent half-life (6.4 vs 3.7 hours). A study of individuals with renal impairment also showed that peak plasma concentration and extent of exposure were higher in these subjects compared with healthy controls5.

In contrast to the absorption, distribution, and excretion of dalfampridine, its metabolism in humans has not been adequately characterized, although the extent of metabolism has been reported to be limited, with approximately 90% of administered drug excreted unchanged in urine6,7. Studies have shown that in rats and dogs there is also limited metabolism of 4-AP, with production of two primary metabolites that were identified as 3-hydroxy-4-AP and 3-hydroxy-4-AP sulfate8. However, an excretion balance study that evaluated the disposition of ^14^C-dalfampridine in humans suggested the presence of the metabolites 2-hydroxy-4-AP and 3-hydroxy-4-AP9. That study also confirmed the rapid and complete excretion by the urinary route, primarily as an unchanged compound with only 0.5% of radioactivity recovered from feces. However, the metabolites, which quantitatively comprised 3% and 6%, respectively, of total radioactivity recovered from urine, were identified based on retention times using high performance liquid chromatography (HPLC) and no appropriate reference standards were used for comparison. Therefore, the objective of the current study was to identify the main metabolites of 4-aminopyridine and to characterize the pathways responsible for 4-AP metabolism in human subjects.

## Methods

### Metabolite identification

Metabolites were identified from plasma and urine samples obtained during an excretion balance study of four subjects (healthy white males, mean age ± standard deviation of 21 ± 2 years) who were administered a single oral 15-mg dose of ^14^C-4-AP (100 μCi) in solution9. Post-dose collection of plasma and urine samples has previously been described9. In the absence of authentic standards during the original analysis, samples were sent to another laboratory (Huntingdon Life Sciences, Cambridgeshire, UK) for more complete characterization. For the current analysis, the urinary profile of metabolites was based on urine samples pooled from the four subjects for the 0–4 hour post-dose period. The plasma profile was based on individual plasma samples from the subjects collected at 2 and 4 hours post-dose, and on plasma samples from 1, 3, and 6 hours post-dose that were pooled across subjects, given the low volumes obtained for the individual subjects at these time points.

Isolation and identification of metabolites using thin layer chromatography (TLC) before and after enzyme hydrolysis with β-glucuronidase (16 hours) and sulfatase (16 and 69 hours), HPLC, and gas chromatography/mass spectroscopy (GC-MS) including electrospray ionization (ESI) were performed as described for 4-AP metabolite characterization in preclinical animal models8. Radioactivity measurement was performed by liquid scintillation analysis using a Wallac 1409 automatic liquid scintillation analyzer (Pharmacia-Wallac Oy, Turku, Finland). Radioactivity in amounts less than twice that of background were considered below the limit of quantitation (BLQ).

Authentic standards of 4-AP, 2-hydroxy-4AP, 3-hydroxy-4AP, and 4-AP-N-oxide (Elan Corporation, Athlone, Ireland) were used in the comparison for identification of metabolites.

### Reaction phenotyping

The internal standard 3,4-diaminopyridine, was purchased from Sigma-Aldrich Corp. (St. Louis, MO), as were ammonium acetate, diethyldithiocarbamate (DDC), dimethyl sulfoxide, ethylene-diaminetetraacetic acid tetrasodium salt (EDTA), furafylline, glucose-6-phosphate, glucose-6-phosphate dehydrogenase, ketoconazole, magnesium chloride, 8-methoxypsoralen, α-naphthoflavone, β-nicotinamide adenine dinucleotide phosphate (β-NADP), phencyclidine, quinidine, sucrose, sulfaphenazole, thio-TEPA, ticlopidine and troleandomycin; potassium phosphate monobasic was from J.T. Baker (Phillipsburg, NJ); montelukast was from Sequoia Research Products (Pangbourne, UK); acetonitrile was from Fisher Scientific (Pittsburgh, PA); and formic acid was from EMD Biosciences (San Diego, CA). 10-Imidazolyl decanoic acid (10-IDA) was a generous gift from Dr. Robert Hanzlik (University of Kansas, Lawrence, KS).

Human liver microsomes were prepared from a mixed-gender pool of 50 individuals. Ascites fluid from mice producing inhibitory monoclonal antibodies against human CYP enzymes were obtained from the National Institutes of Health/National Cancer Institute (NIH/NCI)10,11. Control mouse ascites fluid (lyophilized) was purchased from Cedarlane Laboratories (Hornby, Ontario, Canada). Recombinant human flavin-containing monooxygenase (FMO) enzymes were obtained from BD Biosciences (Woburn, MA) as were recombinant CYP enzymes rCYP2E1, rCYP2J2, rCYP3A4 and rCYP4F2, rCYP4F3a and rCYP4F3b that were co-expressed with cytochrome b5. All other recombinant CYP enzymes that were not co-expressed with cytochrome b5 were obtained from Cypex Ltd. (Dundee, Scotland).

Liquid chromatography coupled with tandem mass spectrometry (LC/MS/MS) was used for the analysis of 4-AP and 3-hydroxy-4-AP. The LC/MS/MS system consisted of two Shimadzu LC-10ADvp pumps (Columbia, MD), a Shimadzu auto sampler (Columbia, MD), a Shimadzu DGU-14A degasser and an API4000 triple quadrupole mass spectrometer (Toronto, Canada) equipped with a Turbo Ionspray ionization source. The Ionspray voltage was set at 1500 kV with the source temperature at 550 °C. LC/MS/MS analysis was carried out with nitrogen as the collision gas. Ion transitions were 95/78 for 4-AP, 111/66 for 3-hydroxy-4-AP, and 110/93 for 3,4-diaminopyridine.

4-AP and 3-hydroxy-4-AP were monitored using isocratic reversed-phase chromatography with a Waters Atlantis HILIC C18 column (150 mm × 2.1 mm, 5 μm column; Milford, MA) preceded by a direct connection Phenomenex C8 guard column (4.0 mm × 2.0 mm; Torrance, CA). The mobile phase was 5 mM ammonium acetate in acetonitrile/water/formic acid at a ratio of 90/10/0.2 (v/v/v). LC separations were performed with an isocratic method at a flow rate of 850 μL/min. The metabolite 4-amino-3-hydroxypyridine was quantified with a calibration curve, and substrate remaining was qualitatively assessed by comparison of analytical response for 4-AP with that of the internal standard.

To determine if the 3-hydroxylation of 4-AP to 3-hydroxy-4-AP was catalyzed by CYP enzymes, 4-AP at 1, 10, and 100 μM 4-AP was incubated for 60 min with human liver microsomes (1 mg protein/mL) in 250 μL of potassium phosphate buffer (50 mM, pH 7.4), MgCl_2_ (3 mM) and EDTA (1 mM, pH 7.4) with and without an NADPH-generating system. Reactions were initiated by the addition of an NADPH generating system (1 mM NADP, 5 mM glucose-6-phosphate and 1 U/mL glucose-6-phosphate dehydrogenase), and were terminated by the addition of acetonitrile containing 3,4-diaminopyridine (50 ng/mL). All incubations were performed in duplicate at 37° ± 1 °C. Precipitated protein was removed by centrifugation (920 *g* for 10 min at 10 °C), and supernatant fractions were analyzed by LC/MS/MS. Zero-time, zero-cofactor, zero-substrate, and zero-protein incubations served as controls.

To establish the incubation conditions under which metabolite formation was proportional to incubation time and protein concentration with <20% substrate consumption, 4-AP (1, 10, and 100 μM) was incubated with human liver microsomes. Conditions included protein concentrations of 0.5, 1, and 2 mg protein/mL at a single incubation time of 60 min, and a single protein concentration of 1 mg/mL for multiple time periods (30, 60, 120, and 240 min). Incubations were performed in duplicate at 37° ± 1 °C in a 96-well plate format with the Tecan Script Time Protein version 1.0.2 on the Tecan Liquid Handling System (Tecan, Research Triangle Park, NC). Incubation mixtures (200 μL) consisted of potassium phosphate buffer (50 mM, pH 7.4), MgCl_2_ (3 mM), and EDTA (1 mM, pH 7.4). Reactions were initiated and terminated as described above. The supernatant fractions of incubations with 100 μM of 4-AP were diluted ten-fold with stopped incubation mixture. Samples were analyzed by LC/MS/MS, with zero-time, zero-cofactor, zero-substrate, and zero-protein incubations as controls.

Michaelis–Menten kinetic constants (K_m_ and V_max_) for the 3-hydroxylation of 4-AP were estimated based on incubations of 4-AP at concentrations of 20, 40, 80, 120, 160, 200, 250, 300, 400, 500, 1000, 1500, and 2000 μM with human liver microsomes (1 mg protein per mL) at 37° ± 1 °C for 60 min. Incubations were performed in 200 μL using 96-well plates as described above. Supernatant fractions were diluted 3-fold with stop reagent and analyzed by LC/MS/MS, with zero-time incubations serving as controls.

Phenotyping was performed by incubating 4-AP (10 μM) with microsomes from individual samples (1 mg protein/mL) to estimate inter-individual differences in metabolite formation for CYP enzymes. Incubations in the presence of direct and time-dependent inhibitors, the latter after a 30-min pre-incubation, were also carried out along with solvent controls. The markers of enzyme activity, as well as their inhibitors are shown in Table 1. Duplicate samples were incubated at 37° ± 1 °C for 60 min in 96-well plates in buffer as previously described. Aliquots of the supernatant fractions were diluted 3-fold with acetonitrile and analyzed by LC/MS/MS; zero-time incubations served as controls. Differences in the rate of 3-hydroxy-4-AP formation were compared with the sample-to-sample variations for the enzyme activities.

**Table 1.** Markers and inhibitors of human microsome enzyme activities.

Enzyme	Marker activity	Inhibitor (solvent)
1A2	Phenacetin O-dealkylation	α-Naphthoflavone (acetonitrile 0.5%) and furafylline* (water)
2A6	Coumarin 7-hydroxylation	8-Methoxypsoralen* (water)
2B6	Bupropion hydroxylation	Phencyclidine* (water) and thio-TEPA* (water)
2C8	Paclitaxel 6α-hydroxylation	Montelukast (acetonitrile 0.5%)
2C9	Diclofenac 4′-hydroxylation	Sulfaphenazole (acetonitrile 0.5%)
2C19	*S*-Mephenytoin 4′-hydroxylation	Ticlopidine* (water)
2D6	Dextromethorphan *O*-demethylation	Quinidine (water)
2E1	Chlorzoxazone 6-hydroxylation	Diethyldithiocarbamate* (water)
3A4/5	Testosterone 6β-hydroxylation	Ketoconazole (acetonitrile 0.5%) and troleandomycin* (acetonitrile 0.5%)
3A4/5	Midazolam 1′-hydroxylation	Ketoconazole (acetonitrile 0.5%) and troleandomycin* (acetonitrile 0.5%)
4A11	Lauric acid 12-hydroxylation	10-Imidazolyl-decanoic acid (water)
FMO3	Benzydamine *N*-oxidation	–

*Time-dependent inhibitor

Using 96-well plates, microsomes (1 mg protein/mL) were pre-incubated with ascites fluid containing inhibitory monoclonal antibodies against CYP1A2, CYP2A6, CYP2B6, CYP2C8, CYP2C9, CYP2C19, CYP2D6, CYP2E1, and CYP3A4 at room temperature for 15 min. The ratio amount of each antibody to microsomes was designed to achieve maximum inhibition. After pre-incubation, the samples were placed on ice, and 4-AP (10 and 100 μM) was added followed by the additions of a solution containing potassium phosphate buffer (50 mM, pH 7.4), MgCl_2_ (3 mM), and EDTA (1 mM, pH 7.4). These samples were then incubated for 60 min at 37° ± 1 °C, after which supernatant fractions were diluted 3-fold with acetonitrile and were analyzed by LC/MS/MS. Incubations containing control mouse ascites fluid served as negative controls.

Incubations of 4-AP (10 and 100 μM) were also performed with recombinant human enzymes rCYP1A1, rCYP1A2, rCYP1B1, rCYP2A6, rCYP2B6, rCYP2C8, rCYP2C9, rCYP2C18, rCYP2C19, rCYP2D6, rCYP2E1, rCYP2J2, rCYP3A4, rCYP3A5, rCYP4A11, rCYP4F2, rCYP4F3a, and rCYP4F3b (25 pmol CYP per incubation) and recombinant FMO enzymes rFMO1, rFMO3, and rFMO5. Since the specific content (pmol CYP/mg protein) among recombinant enzymes varies, recombinant CYP enzymes were normalized with control microsomes. rCYP1B1, rCYP2E1, rCYP2J2, rCYP3A4 and rCYP4F2, rCYP4F3a and rCYP4F3b were co-expressed with cytochrome b_5_ and were normalized with microsomes from baculovirus-infected insect cells containing empty vector to 0.04 mg protein/mL. Incubations with microsomes from baculovirus-infected insect cells containing empty vector served as negative controls for incubations with these recombinant enzymes and recombinant FMO enzymes. rCYP1A1, rCYP1A2, rCYP1B1, rCYP2A6, rCYP2B6, rCYP2C8, rCYP2C9, rCYP2C18, rCYP2C19, rCYP2D6, rCYP3A5, and rCYP4A11 were normalized to 0.05 mg protein per mL with microsomes from *Escherichia coli* containing empty expression plasmid (Control BactosomesTM). Incubations of 4-AP with Control Bactosomes™ and microsomes containing only NADPH-cytochrome *c* reductase (reductase control) served as negative controls for recombinant CYP enzymes not co-expressed with cytochrome b_5_.

Data were processed using Microsoft Excel 2003 (Microsoft Corp., Redmond, WA). To quantify metabolite formation, the line of best-fit was calculated for calibration standards by weighted (1/x) linear regression based on analyte/internal standard (IS) peak-area ratios for two replicates of six calibration standards using Analyst 1.4.1 MS System software (Applied Biosystems/MDS SCIEX, Ontario, Canada). Lineweaver–Burk and Eadie–Hofstee plots (nonlinear regression with appropriate weighting) were used to determine kinetic constants. K_m_ and V_max_ values were estimated using GraFit (version 4.0.21, Erithacus Software Limited, London, UK). Correlation analysis (Pearson product-moment *r* value) was performed with SigmaStat (version 3.1, SPSS Inc., Chicago, IL).

## Results

### Metabolite identification

**Figure 1.**
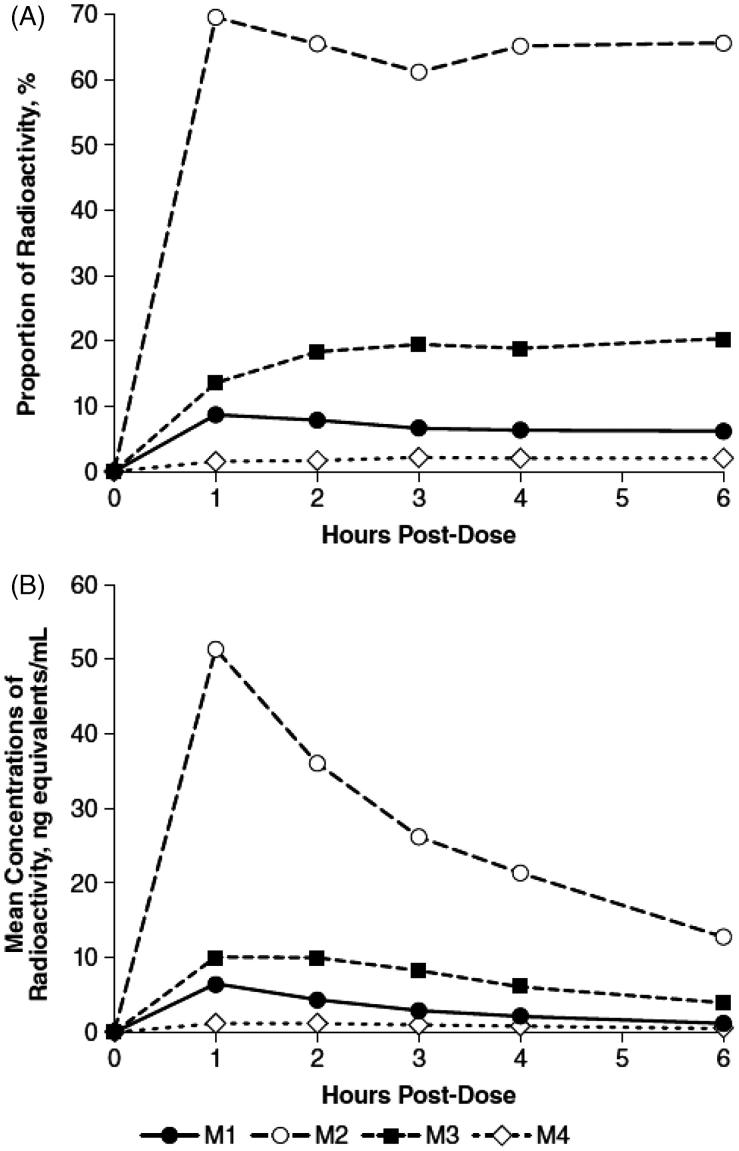
Radioactive components in human plasma determined by thin layer chromatography, expressed as percent of extract radioactivity, after oral administration of 15 mg ^14^C-4-aminopyridine. (A) Mean proportions. (B) Mean concentrations.

Recovery of radioactivity in both urine and plasma was primarily associated with three chromatographic component peaks designated M1, M2, and M3. However, two additional minor components were recovered in plasma, one of which accounted for about 2% of extracted radioactivity (M4), and the other was BLQ although it was visible on the exposed TLC images. While the relative proportions of M2 and M4 in plasma remained nearly constant throughout the collection periods, there was a small decrease in M1 from 8.6% to 6.1% and an increase in M3 from 13.7% to 20.3% (Figure 1A). The calculated plasma concentrations of each of the four components were maximal at 1 h post-dose (Figure 1B), and subsequently decreased over the collection period.

**Figure 2.**
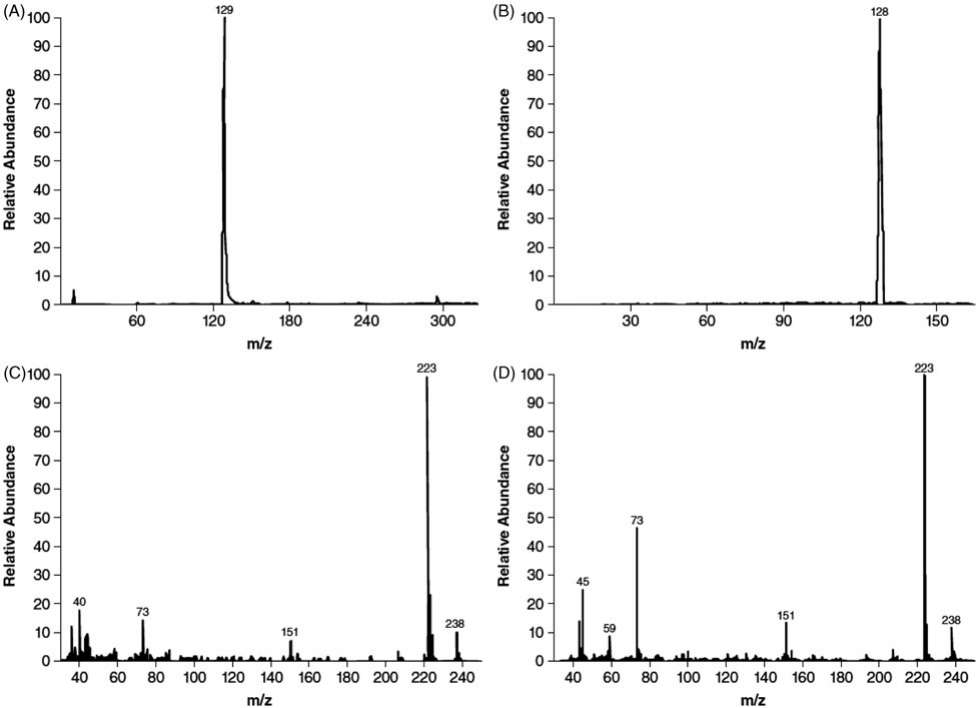
Gas chromatography/mass spectroscopy identification of M2 as 4-aminopyridine. Mass chromatograms of M2 (A) and authentic 4-aminopyridine (B), and mass spectra of M2 (C) and authentic 4-aminopyridine (D). Samples were derivatized prior to gas chromatography mass spectroscopy analysis.

On TLC, the components eluted with R_f_ values of 0.42, 0.59, and 0.75 for M1, M2, and M3, respectively; M1 was identified as 3-hydroxy-4-AP and M2 as unchanged 4-AP based on comigration with authentic reference standards that had R_f_ values of 0.44 and 0.60, respectively. Most of the radioactivity detected in plasma at each time point corresponded to unchanged 4-AP. After derivatization, GC-MS spectra of M2 (Figure 2A) matched those of the trimethyl-silylated derivative of authentic 4-AP (Figure 2B), and mass spectra exhibited a moderately intense ion at m/z 238 and a prominent ion at m/z 223 (Figure 2C) that was also consistent with the derivative of authentic 4-AP (Figure 2D). The derivative of 4-AP presented 6 methyl groups on α-silicon, and therefore the loss of any methyl group gave m/z at 223, the prominent ion in Figure 2(C and D). Further cleavage generated more fragments such as trimethylsilane (m/z 73) and N-(dimethylsilyl)-pyridin-4-amine (m/z 151), which were also observed in our GC-MS analysis in Figure 2(C and D).

**Figure 3.**
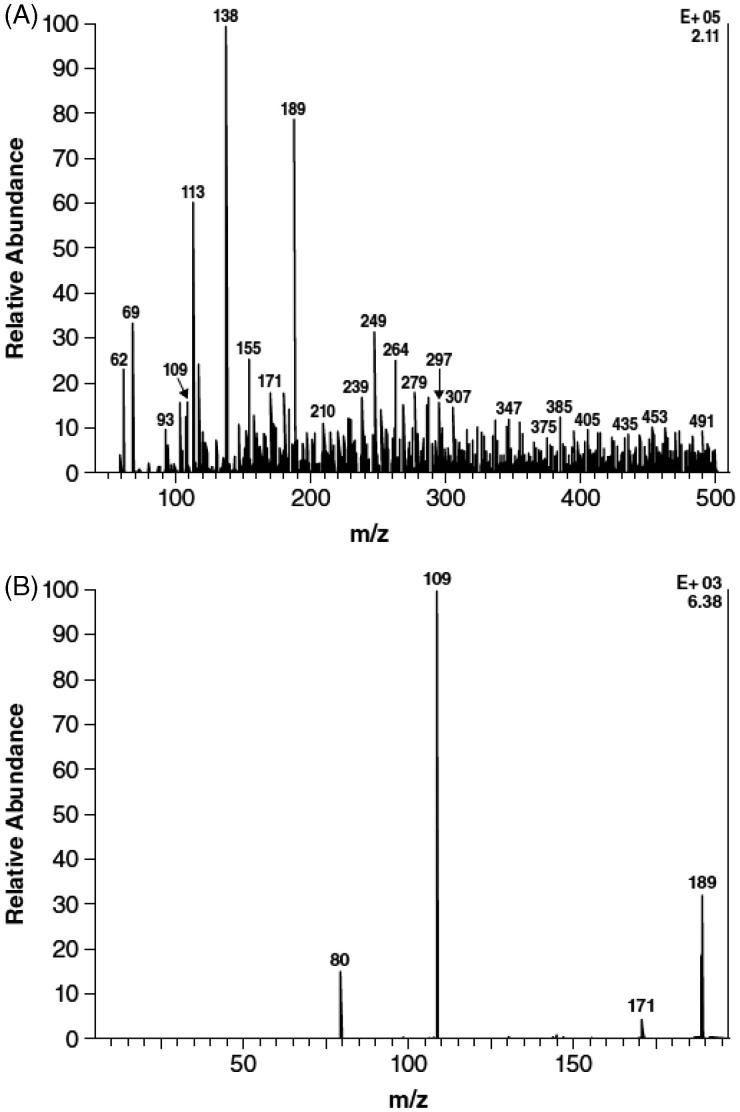
Mass spectra evaluation of M3 isolated from urine after oral administration of 15 mg ^14^C-4-aminopyridine to four human subjects. (A) Precursor electrospray ionization spectrum. (B) Product ion scan of m/z 189 ion of component M3.

Component M3 did not comigrate with any of the authentic standards. However, after treatment of urine with sulfatase, the proportion of M3 decreased with a concomitant increase in the proportion of M1 (3-hydroxy-4-AP) (Table 2). However, the proportion of M3 was unchanged after β-glucuronidase treatment, which contains some sulfatase activity. Component M2 (4-AP) remained unchanged after incubation with the hydrolytic enzymes. A similar reciprocal relationship between the decrease in M3, to levels BLQ, and an increase in M1 after sulfatase digestion relative to untreated plasma was observed in the 2- and 4-hour plasma samples. These data suggest that M3 is a sulfate conjugate of 3-hydroxy-4-AP, a conclusion further supported by negative ESI ionization of M3 that showed a precursor ion at m/z 189 m/z to be one of the strongest signals in the sample (Figure 3A). Subsequent product ion scan obtained by fragmenting the m/z 189 ion (Figure 3B) showed that the molecule appeared to cleave almost entirely into two parts consisting of deprotonated 3-hydroxy-4AP (m/z 109) and 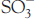 (m/z 80).

**Table 2.** Mean proportions of radioactive components in urine, 0–4 hours post-dose, determined by thin layer chromatography before and after enzyme hydrolysis, expressed as percent of sample radioactivity.

Component	Proportion of sample radioactivity (%)			
	Untreated	β-glucuronidase-treated	Sulfatase-treated	
			16-hour incubation	69-hour incubation
Origin	1.0	1.2	1.4	1.6
M1	4.3	3.9	6.9	7.0
M2	90.3	90.5	90.1	89.1
M3	2.6	2.7	0.4	BLQ

BLQ, below limit of quantitation.

### CYP pathways of 4-AP metabolism

**Figure 4.**
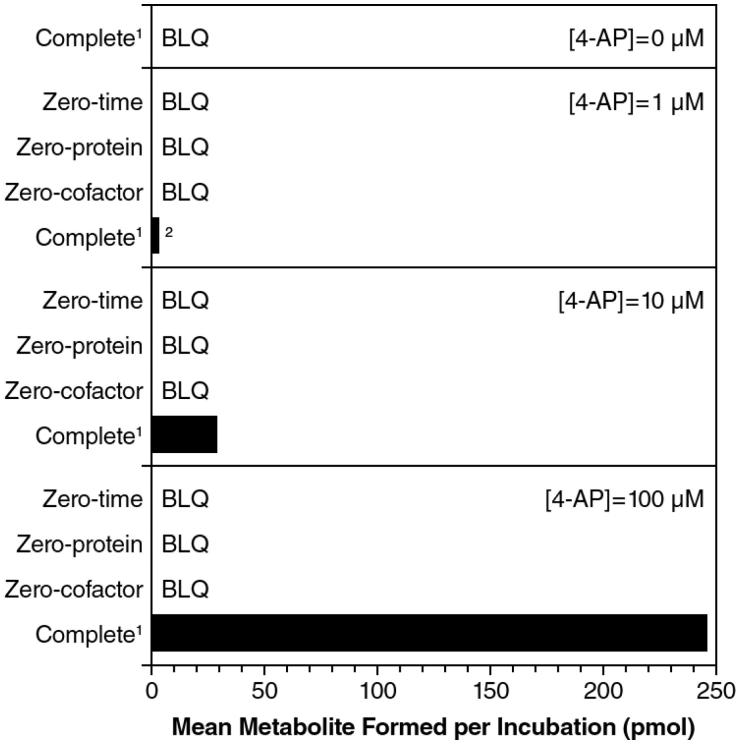
Formation of 3-hydroxy-4-aminopyridine from 4-aminopyridine (4-AP) with incubation of NADPH-fortified human liver microsomes. ^1^1 mg microsomes/mL, 60 min incubations in the presence of NADPH. ^2^Single determination (one sample was below limit of quantitiation). BLQ, below limit of quantitation.

In the initial phenotyping experiments, formation of 3-hydroxy-4-AP from 4-AP was shown to require both the presence of NADPH and microsomal protein (Figure 4), suggesting the involvement of NADPH-dependent enzyme(s) in 4-AP metabolism. At a 4-AP concentration of 1 μM, there was a 3.4% loss of substrate and, at 10 μM, the loss of substrate was 1.7%; there was no measured substrate loss at 100 μM.

Incubation of 4-AP (1, 10, and 100 μM) with pooled human microsomes (0.5, 1, and 2 mg protein/mL) for periods up to 240 min showed that the formation of 3-hydroxy-4-AP increased proportionally with incubation time, protein concentration, and substrate concentration (data not shown). However, while 3-hyroxy-4-AP accounted for all of the substrate conversion to product at the lowest 4-AP concentration, 3-hydroxy-4-AP did not appear to account for all of the substrate conversion as the concentration of 4-AP increased. Based on these data, a crude estimate of K_m_ was determined to be approximately 200 μM, and subsequent experiments were conducted with 1 mg protein/mL for 60 min, which was within the initial rate conditions.

**Figure 5.**
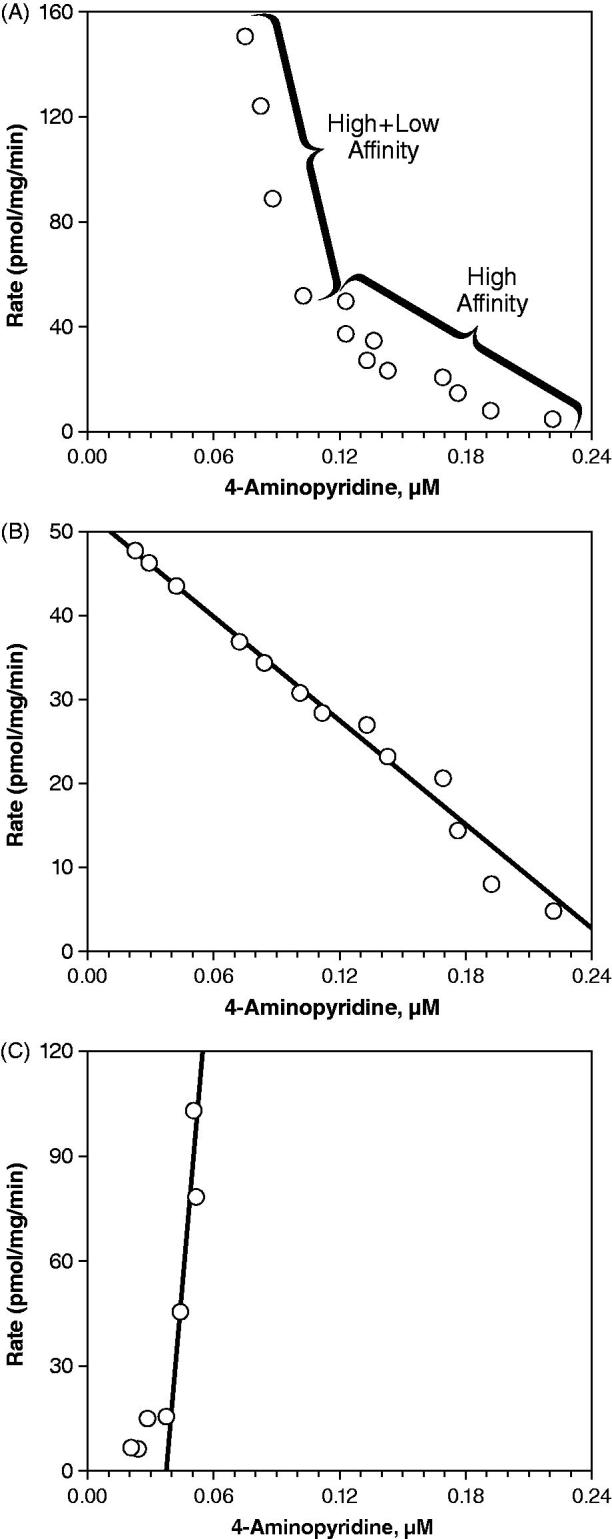
Eadie–Hofstee plots estimating the contribution of human liver microsome (1 mg protein/mL) enzyme(s) to the hydroxylation of 4-aminopyridine to 3-hydroxy-4-aminopyridine. (A) Total enzyme activity. (B) High affinity enzyme activity. (C) Low affinity enzyme activity.

Using the crude estimate for the K_m_, a range of 4-AP concentrations (20 to 2000 μM) was incubated for 60 min with pooled microsomes (1 mg protein/mL) to determine the Michaelis–Menten kinetic constants. The Eadie–Hofstee plot (Figure 5A) revealed a bend, suggesting the involvement of two or more kinetically distinct enzymes in the metabolism of 4-AP. To determine the constants for these kinetically distinct enzymes, separate graphs for the high affinity (Figure 5B) and low affinity enzymes (Figure 5C) were used. For the high affinity enzyme, values of 207 μM and 52.2 pmol/mg/min were estimated for the K_m_ and V_max_, respectively, but kinetic constants could not be determined for the low affinity enzyme, since the values were negative.

In the comparison with the inter-individual variations in activities among the distinct liver samples, the rate of 3-hydroxy-4-AP formation strongly correlated with CYP2E1 activity (*r* = 0.902; *p* < 0.0001), and the intercept of the linear regression line was at the origin, suggesting that CYP2E1 was the primary enzyme for this metabolic transformation. However, the plot also showed that a single sample had CYP2E1 activity that was approximately 5-times greater than the median for pooled microsome samples, and approximately 3.6-times greater than the median 4-AP metabolism rate. Consequently, a second correlation analysis was performed excluding this sample, which revealed moderate correlations with both CYP2E1 (*r* = 0.596; *p* < 0.001) and CYP2C8 (*r* = 0.608; *p* < 0.001). In both cases, the intercept of the linear regression was above the origin suggesting that additional enzymes were involved in the hydroxylation of 4-AP.

With the exception of the antibody against CYP2C9, all of the monoclonal antibodies inhibited the formation of 3-hydroxy-4-AP by more than 20% (data not shown). Consequently, the combined percent inhibition exceeded 100%, and the results were inconclusive.

When metabolism of 4-AP to 3-hydroxy-4-AP was evaluated in the presence of inhibitors, several inhibitors were initially dissolved in methanol at a final concentration of 1%. However, methanol alone inhibited metabolic transformation by approximately 76%, and inhibitors were subsequently dissolved in either water or acetonitrile, of which the latter also inhibited 3-hydroxy-4-AP formation by approximately 33%. All of the direct-acting inhibitors had negligible effects (<20%) at concentrations associated with enzyme-selective inhibition (data not shown), and although quinidine inhibited by 49.5% at 100 μM, it is not selective at this concentration. Although a direct inhibitor of CYP2E1 activity was not evaluated, the CYP2E1 metabolism-dependent inhibitor DDC was evaluated at 5 and 30 μM. At 5 μM, DDC inhibited the formation of 3-hydroxy-4-AP by 41% without pre-incubation and 58% with pre-incubation, and at 30 μM, DDC inhibited the formation of 3-hydroxy-4-AP by 69% without pre-incubation and 77% with pre-incubation. Since the incubation time was 60 min, it is likely that metabolism-dependent inhibition occurred in zero-min pre-incubated samples, resulting in an underestimation of the metabolism-dependent inhibition. When expressed as percent metabolite-dependent inhibition and estimated as the difference between percent of control for zero-min incubation minus the percent of control for the 30 min incubation, none of the other inhibitors demonstrated metabolite-dependent inhibition.

**Figure 6.**
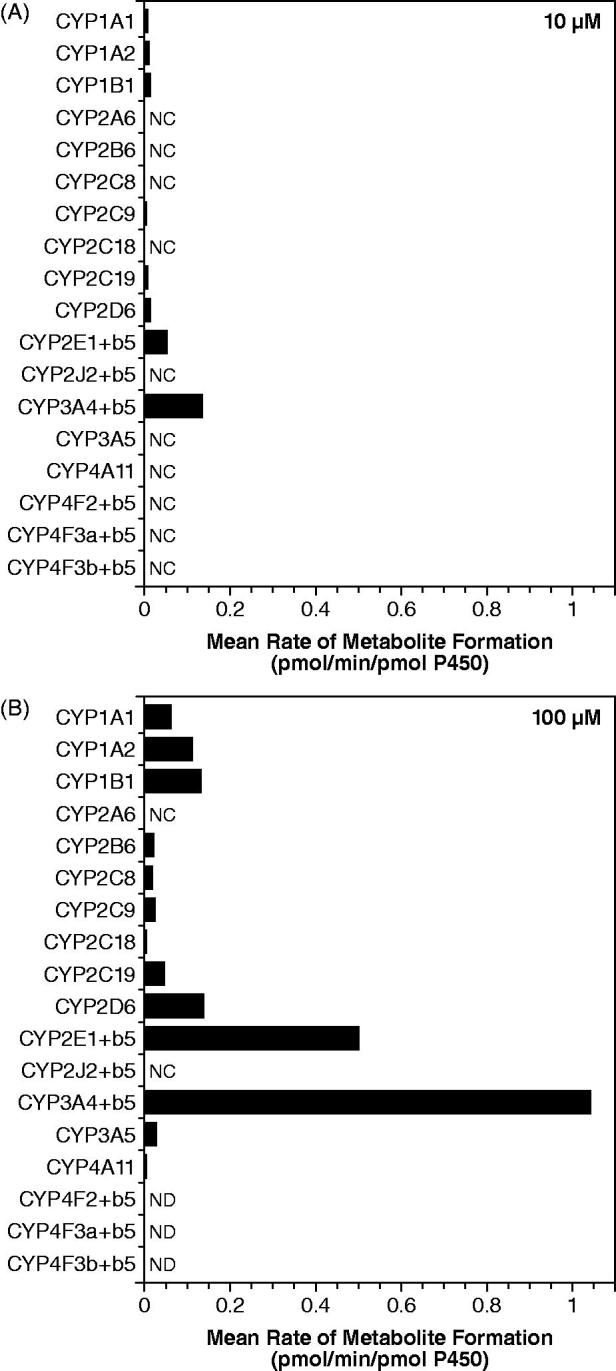
Conversion of 4-aminopyridine to 3-hydroxy-4-aminopyridine after incubation with recombinant human CYP enzymes. (A) 4-aminopyridine concentration 10 μM. (B) 4-aminopyridine concentration 100 μM. Values are blank-corrected with respect to controls. NC, not calculated since no value was detected, values were below the lowest standard, or were less than zero after blank correction; ND, not determined (samples were incorrectly prepared).

When 4-AP (10 and 100 μM) was incubated with recombinant human CYP and FMO enzymes, formation of 3-hydroxy-4-AP was detected in the reductase control and in the insect cell control but not in the Bactosome control; both the reductase control and insect cell control contained reductase activity, while the Bactosome control did not. Consequently, incubations with the recombinant enzymes were blank-corrected using control values to more appropriately estimate the formation catalyzed by each enzyme. Figure 6 shows that while rCYP2E1 catalyzed this conversion (0.0514 pmol/min/pmol P450), rCYP3A4 appeared to be over 2-fold more active than rCYP2E1 (0.134 pmol/min/pmol P450). In addition, several other rCYP enzymes were capable of catalyzing this reaction including rCYP1A1, rCYP1A2, rCYP1B1, rCYP2B6, rCYP2C8, rCYP2C9, rCYP2C18, rCYP2C19, rCYP2D6, rCYP3A5, and rCYP4A11, albeit to a lesser extent than rCYP2E1 and rCYP3A4. Recombinant human FMO enzymes did not catalyze the formation of 3-hydroxy-4-AP.

## Discussion

Characterization of drug metabolic pathways and their metabolites can be important for understanding the efficacy and safety profiles of a pharmacologic therapy, including the potential for drug–drug interactions. The current analysis is the first to specifically focus on characterizing the human metabolites of dalfampridine and determining potential pathways responsible for these metabolites. Data from the two studies reported here confirm that, consistent with what has been reported previously6,7,9, there is limited metabolism of 4-AP in humans. The principal radioactive components recovered from human urine were also present in human plasma and these were identified by TLC and enzymatic digestion with sulfatase as unchanged 4-AP, 3-hydroxy-4-AP, and 3-hydroxy-4-AP sulfate. However, additional minor components that could not be identified were detected in human plasma, M4 and M5, although one of them (M4) only accounted for approximately 2% of the extract radioactivity, and the other (M5) was BLQ.

Most of the radioactivity detected in human plasma during the first 6 hours post-dose corresponded with unchanged 4-AP, which was approximately 8–10 times greater than 3-hydroxy-4-AP and 3 times greater than sulfate conjugate of 3-hydroxy-4-AP during the same time period. However, the proportion of 4-AP in plasma was less than that detected in urine during the 0–4 hour collection period. This further supports that elimination of 4-AP is rapid and consists primarily of unchanged compound. Although the overall proportion of 3-hydroxy-4-AP sulfate was low, it was higher in plasma than in urine; the proportion of 3-hydroxy-4-AP was generally similar in both plasma and urine.

While the proportions of 4-AP and 3-hydroxy-4-AP sulfate in human plasma were similar to those reported in rat plasma, 3-hydroxy-4-AP was notably higher in the human plasma, suggesting that in humans, phase 2 metabolism or elimination of 3-hydroxy-4-AP is less efficient than in rats. The proportion of the unidentified components, M4 and M5, were similar in human and rat plasma, but appear to provide a negligible contribution to the overall metabolic process.

The three major components detected in human urine were the same as those identified in dog urine, although there was proportionately more unchanged 4-AP in human urine (∼90%) than in dog urine (29%)8, suggesting that a substantially greater proportion of 4-AP is excreted as unchanged compound in humans.

Based on these results from the metabolite identification study, it can be proposed that limited metabolism of 4-AP in humans occurs by liver enzymes in a two-step process. The first step consists of hydroxylation of 4-AP to 3-hydroxy-4-AP, followed by conjugation of the 3-hydroxy-4-AP to 3-hydroxy-4-AP sulfate, with slower excretion of the sulfate conjugate relative to 4-AP and 3-hydroxy-4-AP.

Characterization of the pathways involved in metabolic transformation of 4-AP showed that there was very little loss of parent drug, again, consistent with limited metabolism and high excretion of unchanged compound (∼90%). Notably, the metabolism of 4-AP was only measured when the formation of 3-hydroxy-4-AP was specifically monitored. Based on the extent of loss, very little metabolism (∼3.4%) occurred at a 4-AP concentration of 1 μM (94 ng/mL) following a 60 min incubation with NADPH. These results indicate that the overall metabolism of 4-AP *in vitro* is quite slow, consistent with results reported previously in an excretion balance study9.

Although there is only limited metabolism of 4-AP, the initial step in this process, 3-hydroxylation of 4-AP to 3-hydroxy-4-AP by human liver microsomes appeared to be catalyzed by two or more kinetically distinct CYP450 enzymes. At a substrate concentration of 10 μM, which was well below the estimated K_m_ of the high affinity enzyme (K_m_ = 207 μM), CYP2E1 was the major enzyme responsible for the 3-hydroxylation of 4-AP. Identification of CYP2E1 as the primary enzyme responsible for this transformation was consistent in several analyses including correlation analysis, chemical inhibition studies, and incubation with recombinant human CYP enzymes. In particular, DDC was the only metabolism-dependent inhibitor that resulted in substantial inhibition of 3-hydroxy-4-AP formation. However, since the incubation time was long, it is likely that metabolism-dependent inhibition occurred in the zero-min pre-incubated samples, resulting in an underestimation of the metabolism-dependent inhibition with DDC. It should also be noted that the observed inhibition of 3-hydroxy-4-AP formation by methanol further supports CYP2E1 involvement; methanol is a known inhibitor of CYP2E1 activity.

The formation of 3-hydroxy-4-AP by the panel of recombinant CYP enzymes does not provide information on the relative contribution of the individual enzymes to its formation. The recombinant enzymes were used to establish which individual or combinations of enzymes may be involved in the metabolism of 4-AP. These recombinant enzymes differ in their catalytic competency, and are not expressed at concentrations that reflect their levels in human liver microsomes. Therefore, these results can only be interpreted as demonstrating which enzymes may contribute, but not the extent to which each CYP enzyme is responsible for the formation of 3-hydroxy-4-AP.

## Conclusions

In conclusion, there is only limited metabolism of 4-AP in humans, and the two major metabolites present in both plasma and urine were identified as 3-hydroxy-4-AP and 3-hydroxy-4-AP sulfate. Characterization of the metabolic pathways showed that the initial step appeared to be catalyzed by two or more members of the CYP450 family of enzymes. Based on correlation analysis, chemical inhibition studies, and incubations with recombinant human CYP enzymes, CYP2E1 was identified as the major enzyme responsible for this process. The identity of the CYP enzymes suspected of playing a minor role in the 3-hydroxylation of 4-AP could not be established unequivocally. Nevertheless, the metabolic profile of 4-AP in humans suggests not only that there is a low risk for drug–drug interactions via effects on cytochrome-mediated 4-AP metabolic pathways, but the limited and slow rate of metabolism suggests that 4-AP metabolites are unlikely to be actively involved in the pharmacodynamics of 4-AP. However, further evaluation of the potential pharmacodynamic properties of these metabolites is warranted.

## Transparency

### Declaration of financial/other relationship

AC and AB are employees and stockholders of Acorda Therapeutics, Inc., Ardsley, NY, USA.
